# Bidirectional Associations Between Sleep and Alcohol Use in Midlife and Late Adulthood: The Role of Childhood Trauma

**DOI:** 10.1093/geroni/igaf122.3093

**Published:** 2025-12-31

**Authors:** Eunjin Tracy, Jichan Kim, Pei-Shu Chao, Eunjung Kim, Shadi Ansari

**Affiliations:** University of Missouri, Columbia, Missouri, United States; Liberty University, Lynchburg, Virginia, United States; University of Missouri, Columbia, Missouri, United States; University of Missouri, Columbia, Missouri, United States; University of Missouri, Columbia, Missouri, United States

## Abstract

Childhood trauma, including physical, sexual, and emotional abuse, has lasting effects on sleep in adulthood. It may also increase the risk of alcohol use disorders, as individuals use alcohol to self-medicate distress. Sleep disturbances can contribute to increased alcohol consumption, while alcohol use may further impair sleep quality, reinforcing a cycle of disrupted sleep and excessive drinking. However, bidirectional daily associations between sleep and alcohol use remain understudied, particularly in midlife and late adulthood, and the potential moderating role of childhood trauma is unclear. This study analyzed data from 436 participants (M age = 54.11, range = 34–83) in the Midlife in the United States (MIDUS) Biomarker Project, assessing daily fluctuations in sleep quality, sleep efficiency (SE), and alcohol use over seven days, along with retrospective reports of childhood trauma. Individuals with greater childhood trauma exposure reported poorer sleep quality on average. While sleep quality was not significantly associated with alcohol use, SE and alcohol use demonstrated a bidirectional relationship. Better SE predicted lower alcohol use at both the within- and between-person levels. However, at the within-person level, greater alcohol consumption was linked to higher SE, whereas at the between-person level, individuals who drank more on average had lower SE. These findings suggest a complex interplay between sleep and alcohol use. While alcohol may temporarily aid sleep, it could ultimately be associated with poorer SE over time in midlife and late adulthood. Future research should explore the long-term health implications of these daily interactions and inform clinical interventions.

